# A bibliometrics analysis and visualization of autism spectrum disorder

**DOI:** 10.3389/fpsyt.2022.884600

**Published:** 2022-07-18

**Authors:** Ping Rong, Qianfang Fu, Xilian Zhang, Hui Liu, Shuyi Zhao, Xinxin Song, Puxing Gao, Rong Ma

**Affiliations:** ^1^First Teaching Hospital of Tianjin University of Traditional Chinese Medicine, Tianjin, China; ^2^National Clinical Research Center for Chinese Medicine Acupuncture and Moxibustion, Tianjin, China

**Keywords:** autism spectrum disorder, bibliometrics, data visualization, CiteSpace, VOSviewer, COVID-19

## Abstract

**Background:**

The prevalence of autism spectrum disorder (ASD) increased rapidly in the last 20 years. Although related research has developed rapidly, little is known about its etiology, diagnostic marker, or drug treatment, which forces researchers to review and summarize its development process and look for the future development direction.

**Methods:**

We used bibliometrics to analyze papers of ASD in the Web of Science from 1998 to 2021, to draw the network of authors, institutions, countries, and keywords in the ASD field, and visualize the results.

**Results:**

A total of 40,597 papers were included with a continually increasing trend. It turns out that the research on ASD is mainly concentrated in universities. The United States has the largest number of ASD studies, followed by England and Canada. The quality of papers related to ASD is generally high, which shows that ASD research has become a hot spot of scientific research. The keywords of ASD etiology and diagnostic markers can be classified into at least 7 aspects. The detection of keywords shows that ASD research is mostly based on its subtypes, takes children as the study population, focuses on neurodevelopmental imaging or genetics, and pays attention to individual differences. And ASD research has changed greatly under the impact of Corona Virus Disease 2019 in the past 2 years.

**Conclusion:**

We consider the future development direction should be based on the improvement of case identification, accurate clinical phenotype, large-scale cohort study, the discovery of ASD etiology and diagnostic markers, drug randomized controlled trials, and telehealth.

## Introduction

Autism spectrum disorder (ASD) is an early-onset, chronic, and heterogeneous neurodevelopmental disorder characterized by social and communication disorders, a narrow range of interests, and restrictive, repetitive, or stereotyped behavior (1). It includes 4 previous generalized developmental disorders, namely autism disorder, Asperger's syndrome, Heller's disease, and pervasive developmental disorder not otherwise specified (PDD-NOS). Patients usually begin to show symptoms from 6 to 24 months, and some patients grow normally early but appear degenerative changes and loss of language and social skills from 24 to 36 months (2–5). Data collected worldwide in 2001 show that the prevalence of ASD has increased from 0.7 to 1.0% since the 1990s (6), and more data show that its prevalence is gradually increasing in recent years. The study conducted by the Centers for Disease Control and Prevention (CDC) in 11 locations in 2018 shows that there is 1 ASD patient in every 44 8-year-old children (7). The increasing prevalence of ASD may be related to the improvement of knowledge and awareness and the change of the diagnostic criteria, which covers a wider range of diseases. Generally speaking, there are more males than females in ASD patients, and the ratio is about 3–4:1 (8).

Nearly three-fourths of ASD patients have more than 16 medical, mental, or neurological diseases, such as attention deficit hyperactivity disorder, intellectual disorder, self-injurious behavior, and sleep disorder, making it the main cause of mental disability in children under 5 years old (9, 10). In addition, ASD currently lacks specific diagnostic markers and targeted treatment methods, which causes a greater economic burden to individuals, their families, and society (11). In the United States (USA), it is predicted that the annual medical and non-medical costs of ASD will reach 500 billion US dollars by 2025 (12).

Although the prevalence of ASD is increasing gradually, ASD is still one of the frontier problems in scientific research, especially etiology research.

Quantitative analysis of ASD research results, combing the historical development of ASD research, analyzing the research hotspots of ASD, and exploring the high studied etiology and diagnostic markers of ASD have some enlightenment for predicting its research trend. At present, bibliometrics, combining mathematical and statistical methods, can evaluate the current situation and future research trends in a field and can guide the follow-up research work (13). The CiteSpace is an excellent bibliometrics tool, which can intuitively reveal the potential relationship between papers in the form of a scientific knowledge map (14). And the VOSviewer also has advantages in bibliometrics analysis. ASD is the hot spot of current research, and there are some related relevant bibliometric articles. However, previous studies on bibliometrics of ASD mostly focused on a specific field or used a single software (15–17). Therefore, this study used CiteSpace and VOSviewer, combined with bibliometric methods, systematically evaluate the articles on ASD research in the Web of Science (WoS) from 1998 to 2021, focusing on the publishing trend, authors, institutions, countries, published journals, cited articles, research keywords, keywords about etiology and diagnostic markers, and keywords with the strongest citation bursts detection in this research field. This paper quantitatively analyzes the research characteristics of ASD research in the past 20 years, and preliminarily analyzes the research trend of ASD, in order to help the follow-up ASD research work.

## Materials and methods

### Topic research

**Figure 1:**
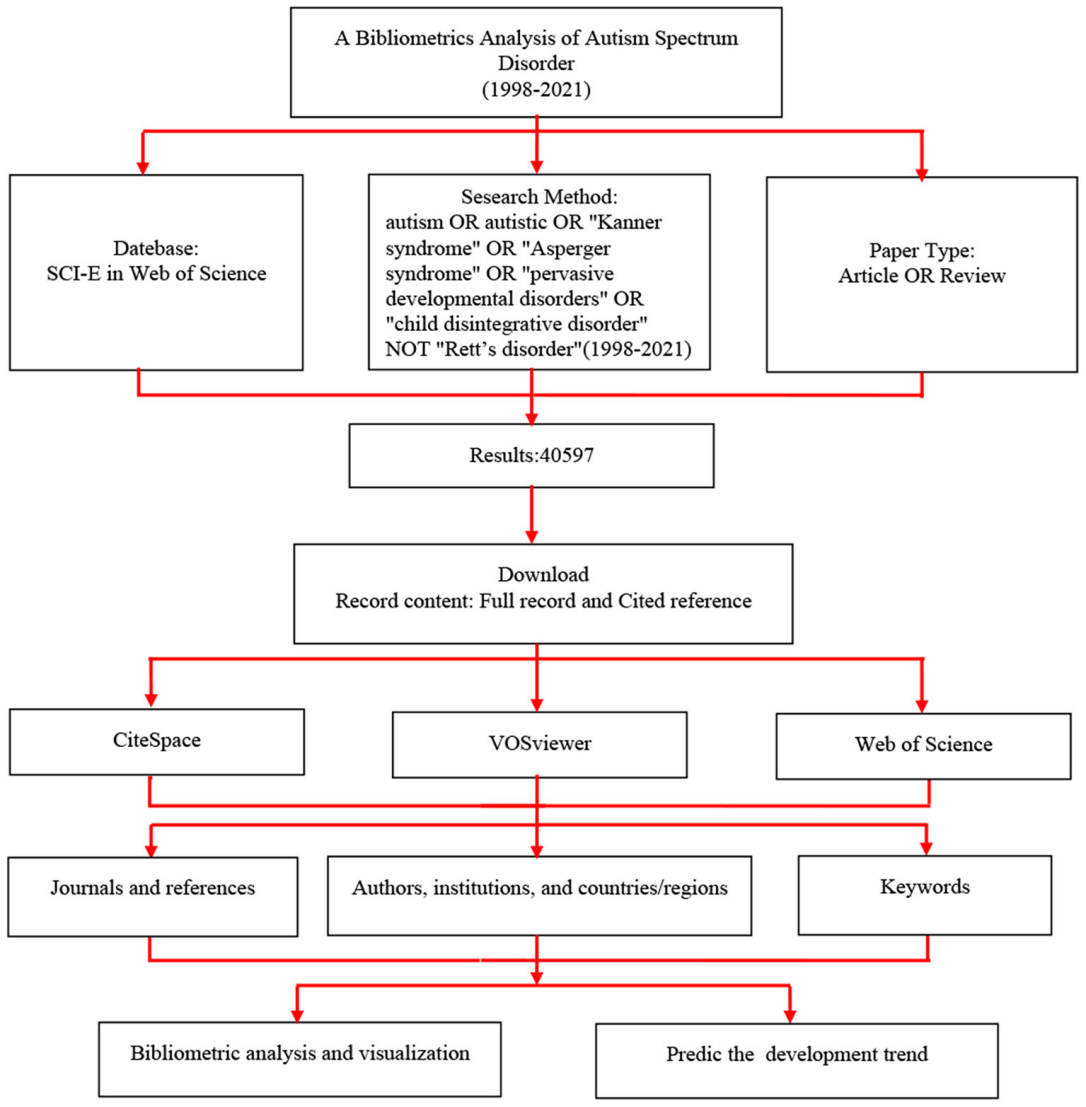
Flow chart of bibliometrics analysis. SCI-E, Science citation index expand.

The article data comes from the science citation index expanded (SCI-E) database on WoS. We searched the WoS with the method: TS = (autism OR autistic OR “Kanner syndrome” OR “Asperger syndrome” OR “pervasive developmental disorders” OR “child disintegrative disorder” NOT “Rett's disorder”), and the time is from the establishment of the database to December 31, 2021 (search date: January 2, 2022). As for the types of articles, we include reviews and articles and exclude meeting abstracts, letters, book reviews, etc. We used the CiteSpace 5.8.R3, and set link retaining factor = 2; Look Back Years = 8; Year Per Slice = 3; Selection Criteria = Top50; Prunning method: pathfinder, pruning sliced networks, and pruning the merged network. We used the VOSviewer 1.6.18 to analyze the keywords, and we selected the first 1,000 keywords as our analysis objectives to study the etiology. And we analyzed the diagnostic markers with the same method. The bibliometrics research process is shown in Figure 1. According to the function of the CiteSpace, the research characteristics of ASD are analyzed from the following perspectives:

1. Analyze the change in the number of articles published in the ASD field over time, which represents the degree of attention in this field to a certain extent;
2. From micro to macro, analyze the authors, institutions, and countries in the field of ASD, which reflects the research characteristics of individuals, institutions, and countries in this field to a certain extent;
3. Analyze the journals and references published in the ASD field, which reflect the quality or attention of research articles in this field to a certain extent;
4. Keywords were carried out to analyze the key research directions in this field since 1998, and we focused on the etiology and diagnostic markers of ASD. Keywords with the strongest citation bursts detection were carried out, which can reflect the changes in research hotspots in a certain period;
5. Based on the above analysis, this paper attempts to predict the trend of follow-up autism research.

## Results

### Annual publishing trends

**Figure 2:**
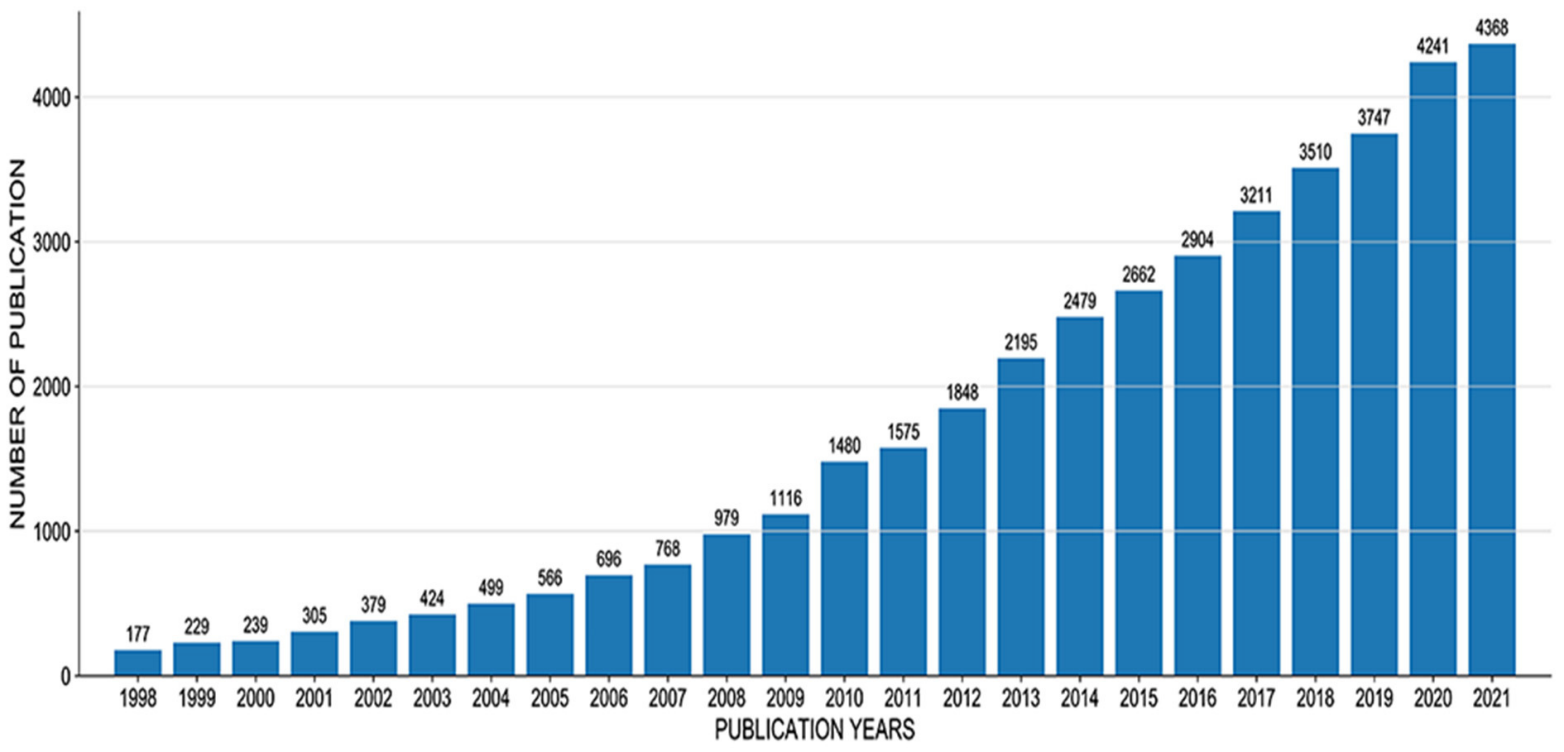
Publication of ASD related papers from 1998 to 2021.

A total of 40,597 articles were included in this study, including 33,489 articles and 7,108 reviews. According to the publication of ASD-related papers (Figure 2), the number of articles on ASD research has increased every year since 177 in 1998 and has risen to at least 4,368 in 2021. The number of papers in this field has increased more than 20 times in the past 20 years.

### Analysis of authors, institutions, and countries/regions

The CiteSpace can analyze the cooperation network among authors, institutions, and countries. In the network, each node represents an individual, the size of the node represents the number of individual papers, the connection between nodes represents the cooperation relationship between individuals, different colors of nodes or connections represent different years, and the color from cold to warm represents the year from far to near. The purple nodes in the outer circle of the network represent the most important nodes, which have centrality > 0.1 (18).

**Figure 3:**
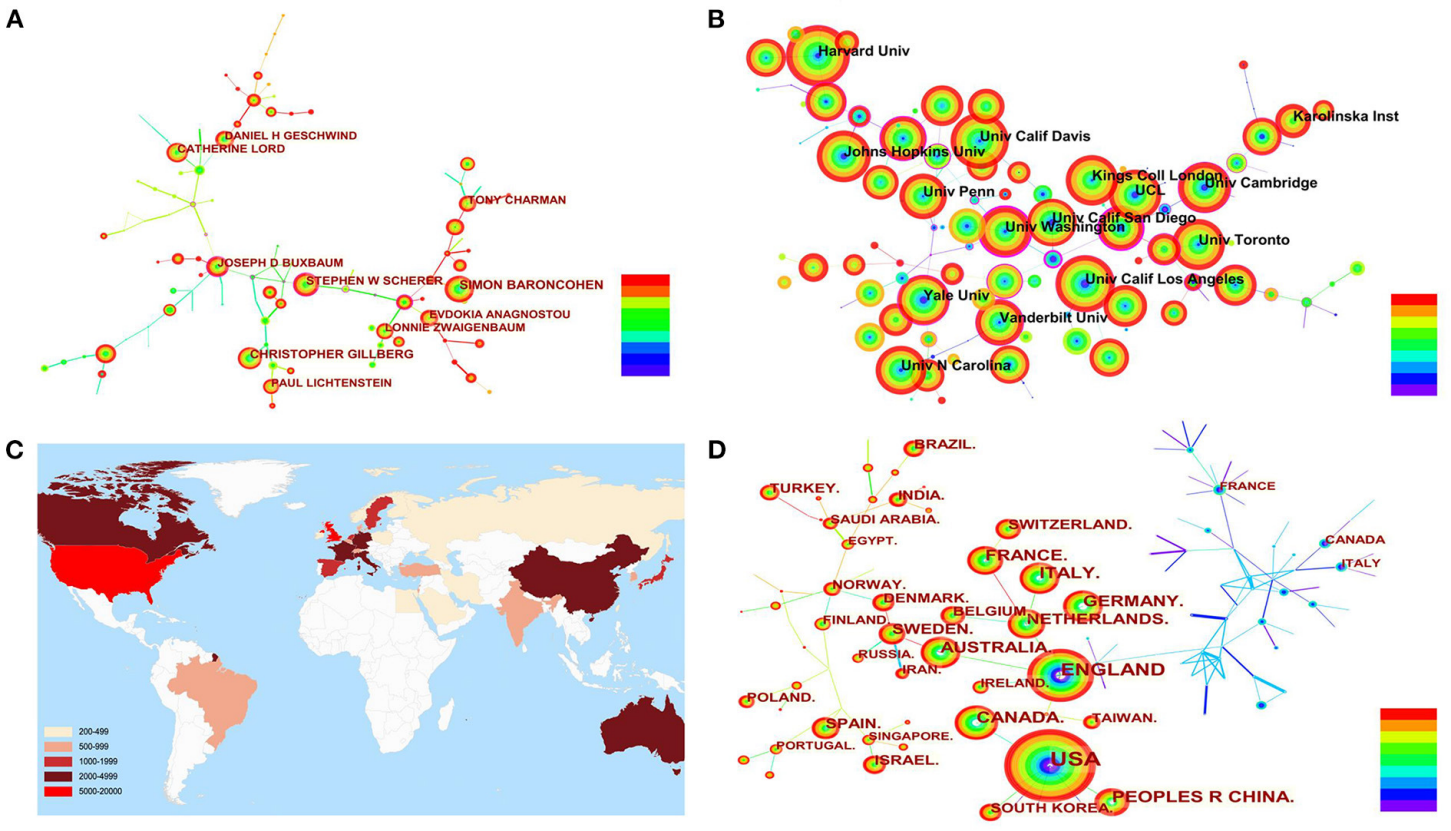
**(A)** Co-cited analysis of authors related to ASD research. **(B)** Co-cited analysis of institutions related to ASD research. **(C)** Global distribution of ASD research (>200). **(D)** Co-cited analysis of countries/regions related to ASD research.

Figure 3A for the cooperative network relationship among authors, which shows the top 10 authors with the largest count of papers. The top 3 authors with the largest number of papers, Simon Baron-Cohen, Christopher Gillberg, and Stephen W Scherer, are from the University of Cambridge, Gothenburg University, and the University of Toronto, respectively.

Figure 3B shows the institutions with the largest count of papers (>600). The institutions in the USA taking the lead in the number of papers included (7/10) and centrality (8/10), followed by England and Canada (Table 1). Among the 19 institutions included, 17 are universities, and only 2 are hospitals with research responsibilities: The hospital for sick children and Kennedy Krieger Institute, which reflects ASD research is still dominated by universities with high basic research instruments and levels.

**Table 1:** The top 10 institutions by count or centrality in ASD research.

**Ranking**	**Top 10 institutions by count**			**Top 10 institutions by centrality**		
	**Count**	**Institution**	**Country/region**	**Centrality**	**Institution**	**Country/region**
1	986	Kings Coll London	England	1.09	Univ Chicago	USA
2	895	Univ Toronto	Canada	0.83	Univ Washington	USA
3	886	Univ Calif Davis	USA	0.81	Univ Oxford	England
4	820	Harvard Univ	USA	0.6	Univ Utah	USA
5	814	Univ Calif Los Angeles	USA	0.47	Brown Univ	USA
6	673	Univ Washington	USA	0.45	Univ Rochester	USA
7	670	Vanderbilt Univ	USA	0.41	Stanford Univ	USA
8	662	UCL	England	0.38	Univ Illinois	USA
9	635	Univ Penn	USA	0.37	Hosp Sick Children	Canada
10	630	Johns Hopkins Univ	USA	0.37	Kennedy Krieger Inst	USA

Figure 3C shows the distribution of countries/regions where articles are published (>200). The USA has the largest number of papers (18,663), followed by England (4,672), and Canada (2,687). Research shows the early research on ASD was mainly in European countries, such as France and Italy (Figure 3D). And the USA published the largest count of articles, but its centrality isn't the highest (centrality = 0.22). Combined with the active research performance of American institutions on ASD, we have reason to believe the USA mostly cooperates among domestic institutions, and the USA has more international cooperation with Asian countries, such as China, South Korea, Singapore, Israel, etc.

### Analysis of journals and references

Table 2 for the top 23 journals with relevant articles published in the ASD field (>250). The impact factor of these journals is generally high, including *molecular psychiatry, biological psychiatry pediatrics, proceedings of the national academy of sciences of the united states of* , *pediatrics, autism research*, etc. The average impact factor is 6.2. Table 3 shows the top 20 papers with the highest citations. These articles focus on the etiology of ASD from the aspects of brain structure, genes, psychology, intestinal flora, and animal model.

**Table 2:** The top 23 journals related to ASD research.

**Ranking**	**Name**	**Count**	**Citations**	**Impact factor (2020)**	**Country/region**
1	Autism Research	1,331	28,723	5.216	USA
2	PLoS ONE	974	30,450	3.2398	USA
3	Scientific Reports	611	7,748	4.38	England
4	Molecular Autism	596	17,051	7.509	England
5	American Journal of Medical Genetics Part A	440	10,301	2.802	USA
6	Molecular Psychiatry	411	27,993	15.992	England
7	Frontiers in Psychiatry	399	3,574	4.157	Switzerland
8	Journal of Child Psychology and Psychiatry	365	19,314	8.982	England
9	Biological Psychiatry	351	31,833	13.382	USA
10	Pediatrics	349	35,382	7.125	USA
11	Translational Psychiatry	347	10,497	6.222	England
12	Behavioural Brain Research	322	13,194	3.332	Scotland
13	Journal of Neurodevelopmental Disorders	302	7,768	4.025	USA
14	Journal of Neuroscience	297	25,469	6.167	USA
15	Journal of the American Academy of Child and Adolescent Psychiatry	296	26,221	8.829	Scotland
16	Proceedings of the National Academy of Sciences of the United States of America	286	32,322	11.205	USA
17	Developmental Medicine and Child Neurology	273	12,130	5.449	England
18	Journal of Developmental and Behavioral Pediatrics	272	9,836	2.225	USA
19	Journal of Child Neurology	267	9,484	1.987	USA
20	Psychiatry Research	262	5,887	3.222	Scotland
21	Frontiers in Neuroscience	260	5,266	4.677	Switzerland
22	Frontiers in Human Neuroscience	255	7,995	3.169	Switzerland
23	Neuroscience and Biobehavioral Reviews	253	18,982	8.989	England

**Table 3:** The top 20 cited articles related to ASD research.

**Ranking**	**The title of article**	**Year**	**Cited number**	**Journal**	**Impact factor**
1	The brain's default network - Anatomy, function, and relevance to disease	2008	6,542	Annals of the New York Academy of Sciences	5.691
2	User-guided 3D active contour segmentation of anatomical structures: Significantly improved efficiency and reliability	2006	4,089	Neuroimage	6.556
3	Dynamic mapping of human cortical development during childhood through early adulthood	2004	3,422	Proceedings of the National Academy of Sciences of the United States of America	11.205
4	Rett syndrome is caused by mutations in X-linked MECP2, encoding methyl-CpG-binding protein 2	1999	3,341	Nature Genetics	38.33
5	A general framework for estimating the relative pathogenicity of human genetic variants	2014	3,213	Nature Genetics	38.33
6	Global burden of disease attributable to mental and substance use disorders: findings from the Global Burden of Disease Study 2010	2013	3,178	Lancet	79.323
7	Understanding and sharing intentions: The origins of cultural cognition	2005	2,168	Behavioral and Brain Sciences	12.579
8	Removing electroencephalographic artifacts by blind source separation	2000	2,105	Psychophysiology	4.016
9	Empathy: Its ultimate and proximate bases	2002	2,096	Behavioral and Brain Sciences	12.579
10	Prevalence of Autism Spectrum Disorder Among Children Aged 8 Years - Autism and Developmental Disabilities Monitoring Network, 11 Sites, United States, 2014	2018	1,919	Mmwr Surveillance Summaries	58.769
11	Strong association of de novo copy number mutations with autism	2007	1,894	Science	47.728
12	Critical periods of vulnerability for the developing nervous system: Evidence from humans and animal models	2000	1,869	Environmental Health Perspectives	9.031
13	Identification of risk loci with shared effects on five major psychiatric disorders: a genome-wide analysis	2013	1,836	Lancet	79.323
14	Psychiatric disorders in children with autism spectrum disorders: Prevalence, comorbidity, and associated factors in a population-derived sample	2008	1,810	Journal of the American Academy of Child and Adolescent Psychiatry	8.829
15	Microbiota Modulate Behavioral and Physiological Abnormalities Associated with Neurodevelopmental Disorders	2013	1,672	Cell	41.584
16	Large-scale brain networks and psychopathology: a unifying triple network model	2011	1,652	Trends in Cognitive Sciences	20.229
17	Building blocks of social cognition: Mirror, mentalize, share?	2019	1,626	Cortex	4.027
18	Consensus Statement: Chromosomal Microarray Is a First-Tier Clinical Diagnostic Test for Individuals with Developmental Disabilities or Congenital Anomalies	2010	1,598	American Journal of Human Genetics	11.025
19	Parvalbumin neurons and gamma rhythms enhance cortical circuit performance	2009	1,559	Nature	49.962
20	Model of autism: increased ratio of excitation/inhibition in key neural systems	2003	1,494	Genes Brain and Behavior	3.449

### Analysis of keywords

#### Analysis of keywords and clusters

**Figure 4:**
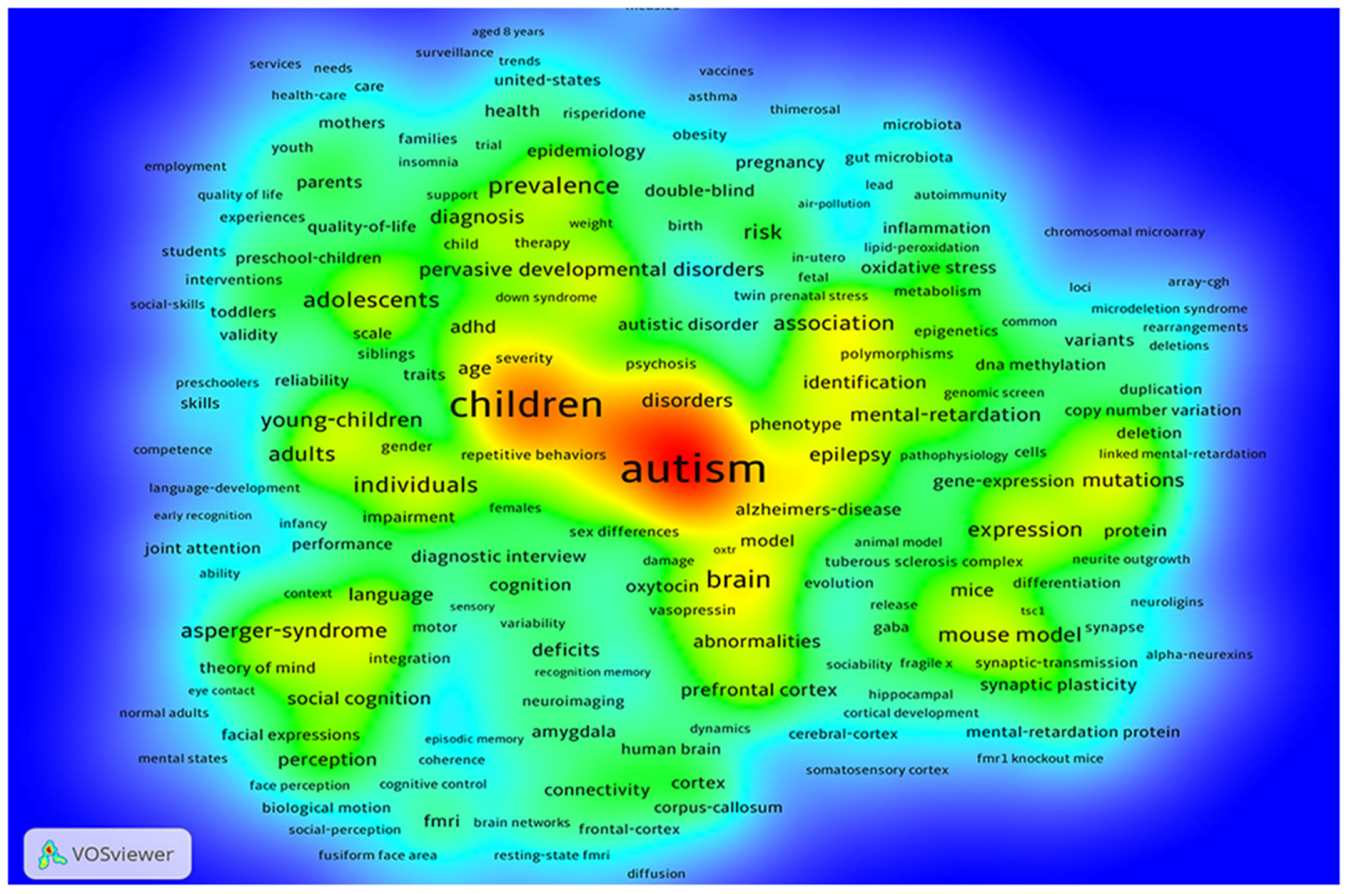
Keyword analysis related to ASD research.

Figure 4 for the keyword density map. Combined with the results, the research population of ASD is concentrated in children, studying with different subtypes of ASD for example fragile X syndrome, Asperger syndrome, or its comorbidities including intellectual disability and insomnia. The ASD research mainly focused on its etiology, including brain structure, image, psychology, genes, as well as animal models. In the research process, the scientists pay attention to the differences between individuals, which is also related to the heterogeneity among ASD individuals.

**Figure 5:**
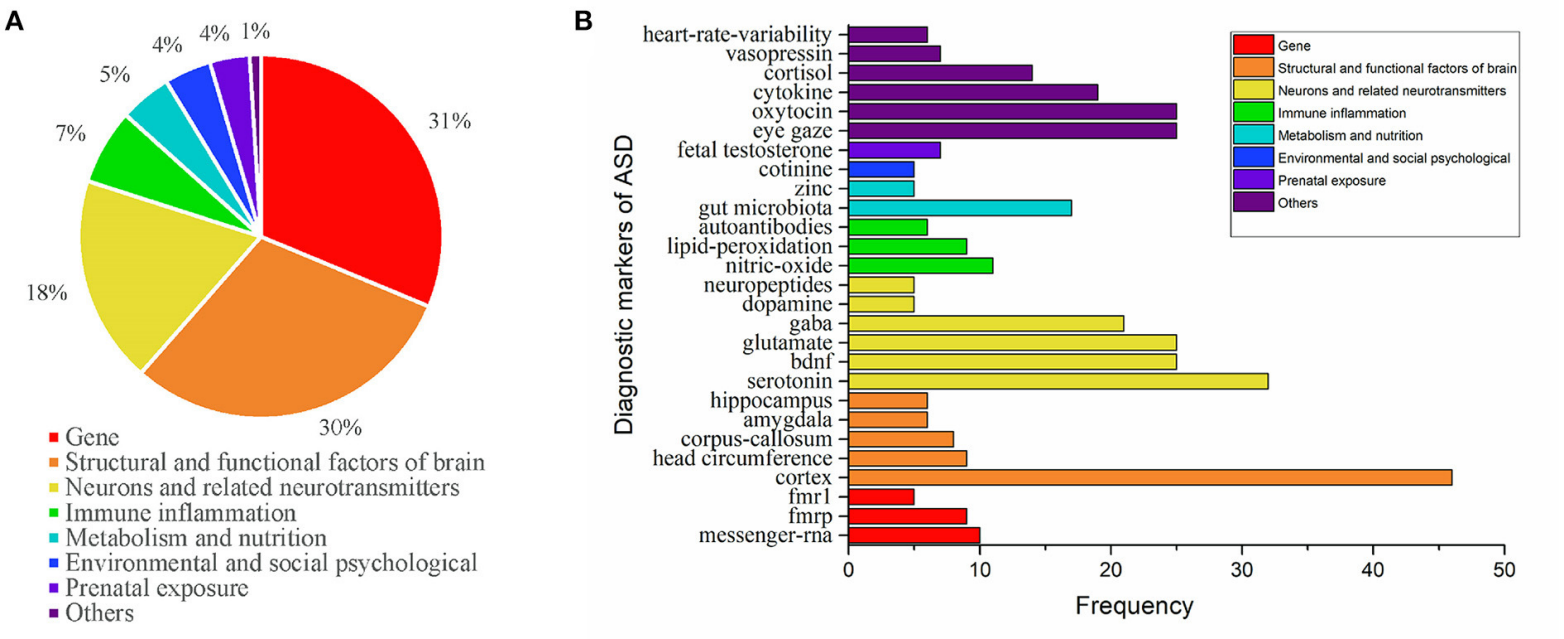
**(A)** Keyword analysis of etiology related to ASD research. **(B)** Keyword analysis of diagnostic markers related to ASD research.

We screened all the keywords and got 239 keywords related to the etiology of ASD. We tried to classify these keywords, and found that the etiology of autism involves genes, structural and functional of brain, neurons and related neurotransmitters, immune inflammation, metabolism and nutrition, environmental and social psychological, prenatal exposure, and others (Figure 5A). In addition, we screened all the literature related to diagnostic markers, and got 858 literature and 366 keywords. And we classify the diagnostic markers according to the etiology (Figure 5B).

#### Analysis of keywords with the strongest citation bursts

**Figure 6:**
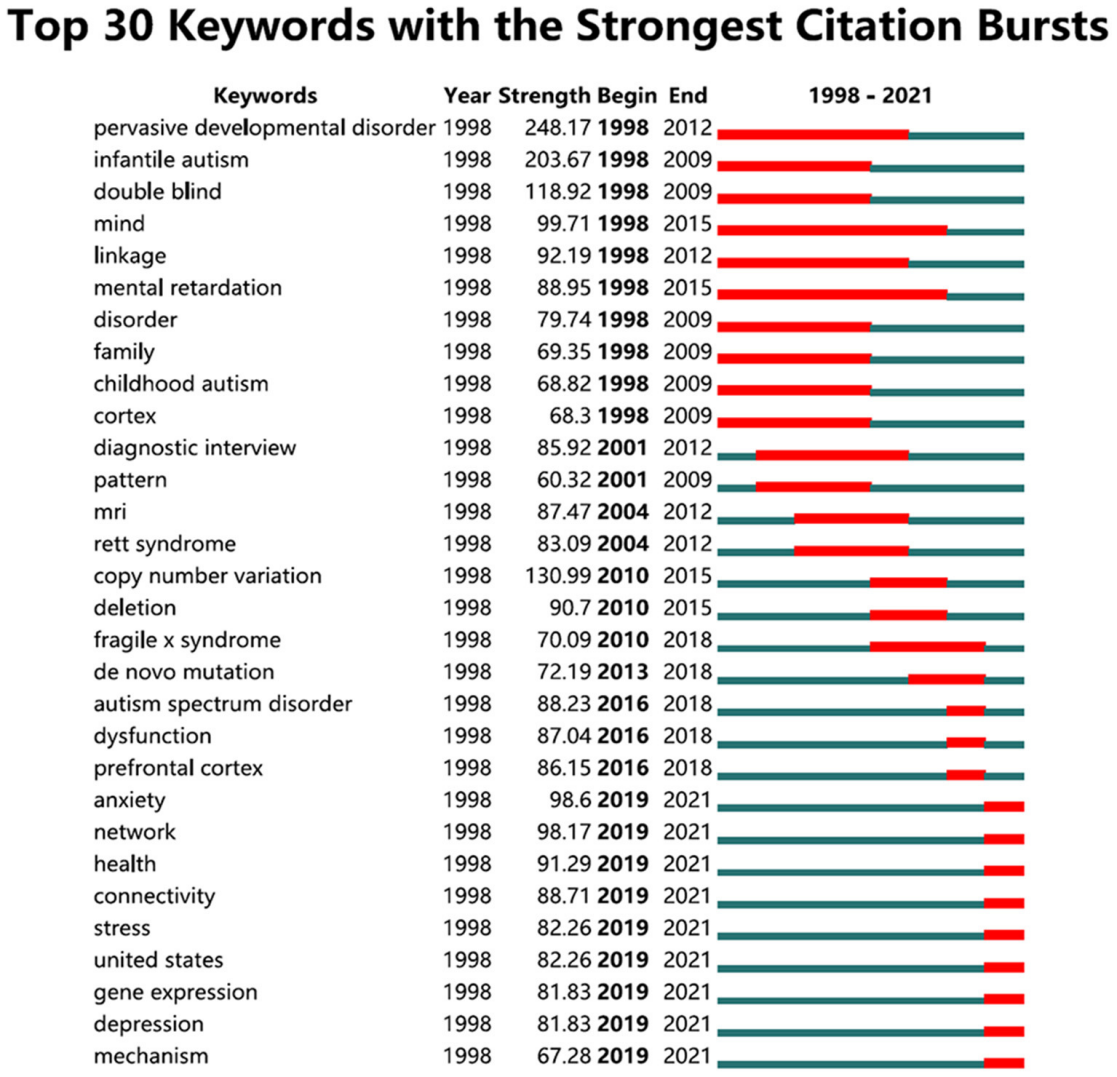
Top 30 keywords with the strongest citation bursts.

Figure 6 for the keywords with the strongest citation bursts, where “Strength” indicates the emergence intensity of keywords, the higher the intensity, the greater the influence, and “Begin” and “end,” respectively, represent the starting and ending years of the keywords. Can be seen from the research on ASD can be divided into three stages: before 2009, 2009–2019, and after 2019. The first stage focused on the etiology of ASD from ASD and its subtypes, neurophysiology, brain imaging, genetic research, and so on. Due to the development of gene technology, the second stage focuses on the research of ASD genes. At the same time, it explored the construction of a variety of animal models. The third stage, because of the influence of Corona Virus Disease 2019 (COVID-19) and the development of detection of intestinal flora, is concerned with the influence of ASD and its guardians under the epidemic situation, and this stage focuses on the relevance between ASD and intestinal flora.

## Discussion

### Temporal and spatial distribution of ASD research

The research papers on ASD have increased by more than 20 times in the past 20 years. There is a large amount of financial support and social attention for ASD. For example, since the 21st century, the USA has promulgated several bills to protect the rights and interests of ASD patients. In 2000, The Children's Health Act of 2000 mandated and the National Center on Birth Defects & Developmental Disabilities was established to promote the etiology, diagnosis, and treatment of ASD. In 2006, the Bush administration promulgated The Combating Autism Act, the first law for ASD patients, and spent $945 million on ASD research, treatment, and education within 5 years, which ended after The Autism CARES Act in 2014. In 2007, Congress promulgated a bill on expanding commitments to ASD patients, proposing to provide $83 million for services for ASD and their families in 2008. From 2007 to 2010, many states in the USA successively legislated to require insurance companies to include the treatment and intervention of ASD into the insurance liability, and some states legislated to make sure ASD patients were protected by the mental health laws on a non-discriminatory basis. In addition, the USA has stipulated the rights and interests of ASD patients in many laws, such as the American with Disabilities Act. The network analysis of countries/regions also shows that the USA has the largest number of outstanding published papers in the field of ASD, which is inseparable from the huge financial support of the USA for ASD. Legal support related to ASD has also been provided in China. For example, the China Disabled Persons' Federation included ASD patients in the category of disabled persons and let them enjoy corresponding rights and interests in 2004. The State Council of the People's Republic of China (SCPRC) stressed the rights and interests of ASD patients to receive education in 2009. The Guidelines for Diagnosis, Treatment, and Rehabilitation of ASD were issued to improve the level of diagnosis and treatment for ASD in China in 2010. In 2014, the SCPRC asked each city to help ASD patients with clinical diagnosis and treatment, infrastructure, and education. For the research on ASD, the National Natural Science Foundation of China has funded more than 3,000 projects since 2010, with a total amount of more than 1.6 billion Chinese Yuan.

In addition to government funding, society and individuals also pay great attention to ASD. For example, in 2002, the Autism Genome Project (19), the largest autism genome research project in history funded by “Autism Speaks” and participated by more than 120 scientists from more than 50 institutes, was launched, and a series of genetic achievements were found. Since the 21st century, the Simons Foundation has also generously funded research on the etiology of ASD, which is a private donation for ASD research. In 2005, *Science* elaborated on the dilemma and frontier of ASD etiology research, which attracted the attention of society and researchers. The famous professor Zheng Yi from China was also invited to interpret it and put forward deep opinions on its future development direction (20). In 2006, Davis, from the MIND Institute at the University of California, launched the Autism Phenotype Project, which is committed to determining the biomedical characteristics of children with different phenotypes through comprehensive and multidisciplinary analysis. In 2007, the General Assembly of the United Nations adopted a resolution to designate April 2 of each year as “World Autism Awareness Day” since 2008, which has attracted extensive international attention to ASD. In 2009, the American Autism Association initiated the global promotion of ASD awareness, scientific research, and services (21). Since 2010, gene detection technologies such as chromosomal microarray analysis have been widely used in ASD Research (22), and gene research on ASD has gradually increased. In 2013, with the promulgation of DSM-5, the new diagnostic criteria for ASD, the further improvement of understanding of ASD, and the increase in clinical prevalence, ASD has further become a research hotspot.

The analysis network of institutions has shown the research of ASD mainly tended to congregate in research universities, and a small number of hospitals participate. The basic research on ASD in the past 2 decades has indeed achieved rich results, but as Professor Laurent Mottron (23) said, we should not be proud of the progress of cognitive neuroscience, genetics, or brain imaging. Our research strategy on ASD needs to be completely changed. The vague diagnosis of DSM-5 makes the included patients have only slight consistency and great heterogeneity, increasing type 2 errors. Therefore, it is suggested that the diagnosis of ASD should be defined by experienced experts too. Professor Zheng Yi also pointed out that the future development direction of ASD is to accurate clinical phenotypes, which need the cooperation of basic research units and units with accurate diagnosis ability, which means more clinicians are needed.

### Keyword analysis

So far, the etiology and pathogenesis of ASD have not been clarified. Our research screened keywords related to the etiology of ASD and found that they generally focus on more than seven aspects, which showed that ASD is a complex multifactorial disease. In the etiology keywords of ASD, the gene, structural and functional of brain, and immune inflammation account for 79%, which represents the degree of concern of scientists to some extent. A meta-analysis showed the heritability of ASD is about 64–91% and a cohort study based in five countries found that the heritability of ASD is 0.8 (24, 25). From the perspective of neurobiology, ASD is regarded as a disease caused by the reorganization of the whole brain in the early stage of development. Children with ASD have abnormal brain development in early life, insufficient overall brain connections, and excessive connections in local areas (often the frontal and occipital regions), which are related to the occurrence of ASD (26). There is also increasing evidence that the central nervous system and a variety of neurotransmitters, such as acetylcholine, serotonin, dopamine, gaba, and glutamate, are associated with the onset and progression of ASD (27).

At present, the early diagnosis of ASD is mainly based on behavioral signs, symptoms, clinical observation, and behavioral assessment. Searching for diagnostic markers related to ASD is of great significance for the early diagnosis and treatment of ASD. We try to classify the selected high-frequency diagnostic marker keywords according to the etiology, and the results have a certain significance. For example, the FMR1 gene and the FMRP protein (product of the FMR1 gene) underlie Fragile X Syndrome, which is the most known genetic monogenic cause of ASD (28). Therefore, the FMR1 gene mutation is recommended as a first-class genetic test in the guidelines (29). Our study found that diagnostic markers focused on cortical studies. Studies found ASD patients had decreased cortical thickness and larger cortical surface areas in several brain regions, including the cingulate, temporal lobes, and amygdala (30). Cortical metabolic abnormalities are also associated with ASD, Kurochkin found that 16 pathways were altered in the prefrontal cortex of ASD subjects (31). Serotonin is also undoubtedly the most studied neurotransmitter related to ASD (27).

According to the detection results of keywords with the strongest citation bursts (Figure 6), the research on ASD in the recent 20 years can be divided into three stages: 1998–2009, 2009–2018, and 2018–2021.

Before 1998: As early as 1926, Grunya Efimovna Sukhareva (32) from the Union of Soviet Socialist Republics had described 6 clinical cases with typical ASD characteristics. More famous is the work of Leo Kanner, the father of child psychiatry. In 1943, Leo Kanner provided detailed medical records of 11 children with social isolation and language disorders. He defined the disease as “autism”, which is derived from the Greek word “autos”, which means “self”, originally used to describe the unrealistic situation of schizophrenic patients. Because ASD is similar to schizophrenia, some people thought the performance of these children was the early performance of schizophrenia. This understanding led to autism being classified as infantile psychosis under the diagnostic umbrella of childhood schizophrenia in DSM-II. It was not until Kolvin's pioneering research in 1971 that error cognition was changed (33). Then, due to the strict limitation of the DSM-III criteria, the diagnostic criteria were expanded in DSM-III-R. In 1944, Hans Asperger (34), an Austrian pediatrician, described children with a social communication disorder, eccentric behavior, abnormal interest, and super functional cognition. His work was later excavated by Lorna wing and named “Asperger's syndrome”, which was fully affirmed in DSM-IV (35). Influenced by ICD-10, DSM-IV published in 1994 named ASD patients with autistic disorder, RTT, childhood disintegrative disorder, Asperger's syndrome, and PDD-NOS. The diagnosis of ASD has been gradually reasonable.

The theoretical explanation of ASD etiology also experienced a long development before 1998. In 1949, Kanner described the parents of children with ASD as compulsive, perfectionist, and lacking a sense of humor. He pointed out vaguely that the severe stress suffered in the early stage, such as parental discord, separation, or mothers' depression, is related to autism. Bruno Bettelheim also believed that autism was not born but caused by the mothers' estranged based on his experience in Nazi concentration camps (36). This theory of attributing the etiology of ASD to parental rearing is absurd at present, but it was widely popular at that time. This theory was overthrown in the 1970s. DeMyer et al. (37) found that there was no difference between parents of ASD children and normal children in terms of infant rearing, feeding, tactile, or general stimulation. When it was found that maternal deprivation was not the cause of ASD, researchers turned to construct new interpretation models including emotional, cognitive, and intersubjective models. Among them, the cognitive model is relatively more concerned for researchers, such as the theory of mind. Theory of mind is an ability to explain the psychological state of themselves and others, which allows individuals to consider and reasonably explain the behavior patterns of others. However, in ASD individuals, the asymmetry between their own knowledge and others' knowledge is often found (38). This is why the patients with ASD perform poorly in the theory of mind tasks (39). The theory of mind combined with the “social brain” theory proposed by Brothers (40), has made great progress in the follow-up ASD research.

1998–2009: Since Leo (41) put forward the concept of “early-onset infantile autism” and the improvement of a series of criteria such as DSM-II, DSM-III, DSM-III-R, and DSM-IV, the research on ASD and its subtypes has become a lasting trend. DSM-IV and ICD-10 use “Pervasive Developmental Disorders” to name ASD, which includes five subtypes, and the research on these subtypes has also continued to become a hotspot. The etiology research of ASD focused on developmental neuroimaging and genes. As early as Leo's article, there was a description of an abnormal increase in head circumference of autistic children. Since then, research around head circumference has also been a hotpot. For example, some researchers (42) found that the head size at birth of ASD patients is smaller than that of normal infants, but the head size suddenly increases excessively between 1–2 months and 6–14 months after birth, and this phenomenon of abnormal growth acceleration may be an early warning signal of ASD risk, which provides a certain reference for the early identification. Some researchers (43) found that this abnormal brain expansion phenomenon mainly occurs in boys with behavioral degradation, rather than in girls, which is consistent with the sex difference of ASD. There are also studies on the function of brain regions combined with PET, fMRI, EEG, etc. For example, as early as 1990, Brothers (40) put forward the “social brain” theory, he thought there are special areas in the primate brain to recognize and understand others' expressions, including the orbitofrontal cortex, superior temporal gyrus, and amygdala. Follow-up researchers carried out research on ASD based on social brain theory, for example, PET (44) was used to observe the changes in the medial prefrontal cortex, temporal-parietal junction, and superior temporal sulcus of temporal pole in children with ASD. It was found that the activation of these areas was less than that in the normal group when completing social tasks. MRI was used to scan the brains of children with ASD and the control group, it was found that the volume of the amygdala and the connection between the amygdala and cortex were related to ASD (45). Some researchers have also found that ASD is related to the early development failure of the mirror neuron system (46). The mirror neurons in children with ASD have structural and functional abnormalities (47, 48), and then put forward the broken mirror theory. Compared with the maturity and wide application of gene technology in recent 10 years, gene research around ASD before 2010 is relatively scarce at this stage, but it has also produced many outstanding achievements, for example, the gene research around RTT. Because RTT mainly occurs in women, they proposed that RTT is mainly caused by X-linked dominant mutation and is fatal in hemizygous males (49). There are also breakthroughs in gene research around RTT including the correlation between the MECP2 gene and RTT (50). In addition, this stage also focuses on ASD caused by family inheritance.

2010–2018: This stage is different from the research before 2010 in all aspects. The generalization of new ASD diagnostic criteria in 2013 reduced the research on ASD subtypes to a certain extent, which can also be seen in Figure 6. At this stage, gene detection technologies such as chromosome microarray (CMA) are widely used in the field of ASD. For example, the expert consensus was formed in 2010 which strongly supported the use of CMA instead of G-band karyotype analysis as the primary cytogenetic diagnostic test for patients with developmental delay/intellectual disability, ASD, or multiple congenital anomalies. For patients with obvious chromosome syndrome (such as Down syndrome), family history of chromosome rearrangement, or multiple abortions, G-band karyotype analysis should be retained (22). From chromosome rearrangement to copy number variation to whole-exome sequencing, gene research has become a feature of this stage. The Autism Speak summarizes the top 10 research progress in the field of ASD every year, and gene research can always be seen. The internationally renowned “autism genome project”, a large-scale cooperative genetics research project (19), initiated by the National Alliance for Autism Research and the National Institutes of Health, has carried out research on the ASD genome and derived achievements since 2010 (51–54). It focuses on the identification of autism susceptibility genes, the genetic mechanism of phenotypes, and the genetic correlation of sex differences and contributed a lot. The results of genetic research on ASD are also outstanding. For example, the cohort study based in five countries found that the heritability of ASD is 0.8 (25). It was found that more than 100 ASD risk genes with a rare and exogenic mutation in about 10–25% of patients (55), and more than 800 genes are considered to play a role in ASD (56). However, some researchers (57) believed that there is insufficient evidence of these genes as ASD specific genes in follow-up studies, because these genes are also related to other clinically defined brain diseases, for example, intellectual disability, epilepsy, schizophrenia, and also overlap with the phenotypes of many genetic syndromes, such as Down syndrome, tuberous sclerosis, angleman syndrome, etc.

After 2019: ASD patients usually suffer from comorbidities including insomnia, anxiety, depression, attention deficit hyperactivity disorder, self-injury behavior, etc. (58). During the COVID-19 outbreak, home isolation caused a certain degree of psychosocial and behavioral problems for ASD children previously diagnosed, such as more serious and more frequent destructive behaviors (59). At the same time, it also exacerbated the anxiety, depression, and social pressure on their caregivers. Amorim et al. (60) found the average anxiety score of parents with ASD children (8.75 ± 0.96) was significantly higher than that of parents with healthy children (5.36 ± 2.71) during the COVID-19 pandemic. Alhuzimi (61) conducted an online questionnaire on 150 parents of ASD children in Saudi Arabia and 94% of parents said that their stress level increased, while 78.7% of parents said that the pandemic had a negative impact on their emotional health. Lugo-Marín (62) observed an increased negative emotion in parents according to the data collected 8 weeks after the beginning of the blockade in Spain, including obsessive-compulsive behavior, depression, anxiety, aggression, etc.

ASD patients generally have gastrointestinal symptoms such as diarrhea, abdominal distension, abdominal pain, and constipation (63), and these symptoms may be related to the imbalance of intestinal flora in ASD patients (64, 65). More and more studies have shown that there is an obvious imbalance of intestinal flora in children with ASD (5, 66, 67). A large number of studies have confirmed that the changes in intestinal flora have a certain correlation with the occurrence of ASD. Intestinal flora can affect brain development through the microbial gut-brain axis, including the metabolite pathway (68), immune pathway, vagus pathway (69), and neuroendocrine pathway (70), which affect the brain function and behavior of patients. However, a study of the intestinal flora of ASD (71) found limited evidence that ASD is directly related to the microbiome of the intestinal flora, and the reason why ASD is related to the intestinal flora may be that ASD-related characteristics promote restrictive dietary preferences, resulting in reduced microbiome diversity.

The treatment of ASD is still based on education and behavior training, but there are many candidate drugs for the treatment of ASD in recent years. In 2010, Lemonnier and Ben-Ari (72) reported for the first time that bumetanide could reduce autism behavior in ASD children without adverse reactions. Although a number of randomized controlled trials (RCTs) about bumetanide in recent 10 years are contradictory, a meta-analysis shows that bumetanide can significantly improve the severity of ASD symptoms including the social effect, and the repetitive patterns of behavior, interests, or activities (73). Although there were adverse reactions such as mild polyuria/polyuria and mild hypokalemia, which could be relieved without additional treatment. The study with the permission of the Israeli Ministry of Health (74), on the use of cannabis diphenol in 53 children showed that it could significantly improve the related and combined symptoms of ASD children. The nasal oxytocin has optimistic application prospects despite disputes (75–77). Due to the widespread nutritional deficiency and metabolic imbalance in ASD, some researchers focused on the supplements of nutrition or change of diet in the treatment of ASD, which includes vitamin D, omega-3 fatty acid, folinic acid, probiotics, coenzyme Q10, gluten free diet, etc. However, the evidence for the relation between supplements nutrition or change of diet and ASD symptoms in childhood is generally insufficient (78). Other promising treatments include fecal microbiota transplantation, hyperbaric oxygen, Tomatis sound, etc. (79, 80).

## Conclusion

Since Leo Kanner published the case report on early-onset infantile autism in 1943, ASD research has a history of more than 70 years. Although we still know little about its etiology, diagnostic markers, and drug treatment, the increasing ASD research and the introduction of new research methods such as gene research and intestinal flora still make the ASD research develop gradually and have a promising prospect. Combined with the development track of ASD research and the analysis of CiteSpace, we believe that ASD will have such a development trend in the future:

a. Improvement of case identification and accurate clinical phenotype: there is no doubt that the diagnostic criteria of ASD are gradually improved from DSM-II to DSM-5. ASD is a pedigree disease rather than a continuum of severity is undoubtedly the result of an in-depth understanding of ASD, which leads to more cases being included in the clinic. However, this diagnosis increases the heterogeneity of the ASD population to some extent, so it also hinders the study of ASD. At present, some researchers (81) try to identify meaningful subgroups in ASD, and it seems still out of reach to find ASD subgroups at the biological level. The suggested methods are as follows: identifying cases by experienced clinicians (23), adding additional specifiers to the original DSM criteria to add subgrouping (82), or familial ASD and non-familial ASD will be studied separately (83).
b. Carry out cohort research in combination with genomics and neuroimaging: it is necessary to prospectively adopt larger-scale sequencing technology, behavior, and MRI research for high-risk people with ASD with early medical risk (such as premature birth) or specific genetic background. For example, the Autism Phenotype Project. These measures are of great significance for the study of its etiology and diagnostic markers.
c. Treatment: Although there are many disputes about ASD candidate drugs such as oxytocin and bumetanide, as Professor Daniel pointed out (77), studies on changing age, dosage form, or course of treatment, as well as larger RCTs still make these candidate drugs have optimistic research prospects. At the same time, we should note that about half of ASD patients use complementary and alternative medicine (CAM), including dietary supplements, herbal therapy, acupuncture, neurofeedback, music therapy, and others. And the utilization rate of CAM in ASD patients is higher than that in other mental diseases and the general population (84). Researchers should also study its effectiveness and safety.
d. Telehealth: In view of the influence of the COVID-19 on ASD patients and caregivers, it is necessary to consider continuous isolation management or public health emergencies. When telehealth becomes the necessary choice, we need to objectively evaluate its reliability, effectiveness, and acceptability in ASD diagnosis and treatment guidance.

## Limitation

This study conducted a comprehensive bibliometric analysis of articles in the field of ASD and analyzed the changes in ASD research in three stages. However, this study has some limitations that need to be solved. Firstly, Because of the limitations of the software, we only analyze English articles in the WoS database, which may lead to language and publishing bias. Secondly, the software is mainly used for statistics and visualization, and our discussion on the future may be insufficient.

## Data availability statement

The original contributions presented in the study are included in the article/Supplementary Material, further inquiries can be directed to the corresponding author/s.

## Author contributions

PR and RM contributed to the conception and design of the study. XZ, HL, and SZ collected data from the database. PR, QF, and XS wrote the first draft of the manuscript. PR, QF, and PG wrote sections of the manuscript. All authors contributed to manuscript revision, read, and approved the submitted version.

## Funding

This work was supported by the National Natural Science Foundation of China (No. 82174439) and the Program for Qi Huang Scholar (RM) in State Administration of Traditional Chinese Medicine.

## Conflict of interest

The authors declare that the research was conducted in the absence of any commercial or financial relationships that could be construed as a potential conflict of interest.

## Publisher's note

All claims expressed in this article are solely those of the authors and do not necessarily represent those of their affiliated organizations, or those of the publisher, the editors and the reviewers. Any product that may be evaluated in this article, or claim that may be made by its manufacturer, is not guaranteed or endorsed by the publisher.
